# Qualitative research within trials: developing a standard operating procedure for a clinical trials unit

**DOI:** 10.1186/1745-6215-14-54

**Published:** 2013-02-21

**Authors:** Frances Rapport, Mel Storey, Alison Porter, Helen Snooks, Kerina Jones, Julie Peconi, Antonio Sánchez, Stefan Siebert, Kym Thorne, Clare Clement, Ian Russell

**Affiliations:** 1 College of Medicine, Swansea University, Singleton Park, Swansea SA2 8PP,UK; 2 Department of Medicine, Cardiff University Llandough Hospital, CF64 2XX, Penarth, UK

## Abstract

**Background:**

Qualitative research methods are increasingly used within clinical trials to address broader research questions than can be addressed by quantitative methods alone. These methods enable health professionals, service users, and other stakeholders to contribute their views and experiences to evaluation of healthcare treatments, interventions, or policies, and influence the design of trials. Qualitative data often contribute information that is better able to reform policy or influence design.

**Methods:**

Health services researchers, including trialists, clinicians, and qualitative researchers, worked collaboratively to develop a comprehensive portfolio of standard operating procedures (SOPs) for the West Wales Organisation for Rigorous Trials in Health (WWORTH), a clinical trials unit (CTU) at Swansea University, which has recently achieved registration with the UK Clinical Research Collaboration (UKCRC). Although the UKCRC requires a total of 25 SOPs from registered CTUs, WWORTH chose to add an additional qualitative-methods SOP (QM-SOP).

**Results:**

The qualitative methods SOP (QM-SOP) defines good practice in designing and implementing qualitative components of trials, while allowing flexibility of approach and method. Its basic principles are that: qualitative researchers should be contributors from the start of trials with qualitative potential; the qualitative component should have clear aims; and the main study publication should report on the qualitative component.

**Conclusions:**

We recommend that CTUs consider developing a QM-SOP to enhance the conduct of quantitative trials by adding qualitative data and analysis. We judge that this improves the value of quantitative trials, and contributes to the future development of multi-method trials.

## Background

Pragmatic randomized trials are common within clinical research. When well designed and applied, they provide methodologically robust ways of investigating the clinical and cost effectiveness of treatments, interventions, or other aspects of healthcare provision [1]. In recent years, health-service researchers, commissioners, and users of research findings have increasingly recognized the value of including qualitative components in research [2,3]. Qualitative methods add to our understanding of complex social worlds, asking questions about the manner in which people behave and communicate, and their understanding of the world and their place within it. Qualitative components of trials serve several purposes including: developing research hypotheses and instruments; gathering complementary information to contribute to answering research questions in depth; helping to explain findings; and understanding whether interventions can and should be implemented, by assessing their acceptability to service users and healthcare professionals. Qualitative work may also add to our understanding of trial design, research methods (including recruitment), data analysis, and reporting. Qualitative methods may be used at all stages of trial development and reporting. They can be particularly helpful in developing and evaluating complex interventions [4].

### Integrating qualitative and quantitative methods and outputs in trials

In clinical trials that do include qualitative components, methods of data collection and analysis, and of sampling strategies are often unclear or not even described. There are methodological gaps and failures in integrating qualitative and quantitative findings [5]. In particularly, methodological gaps and integration problems occur around the inclusion of appropriate methods of data collection that should be influenced by the relevant theoretical paradigms at play. In qualitative terms, the theoretical basis to the trial work influences not only the choice of data-collection method, but also the data-analysis approach, and the theory should be linked to both topic area and research questions. Theory and practice should go hand in hand, including in trials work. For example, if researchers wish to examine a patient group’s ‘lived experiences’ of a particular problem, they may wish to pursue a phenomenological approach at both methodological and paradigmatic levels.

The trials qualitative researcher (TQR), who is employed to support the chief investigator (CI), within a trial would need to discuss both the methodological approach and paradigm with the CI, advising how best to undertake, in this case, phenomenological data capture and analysis, and on which phenomenological paradigm to employ (for example, descriptive or hermeutic phenomenology, aligning with phenomenological interviewing techniques [6,7]). To fit within a trial most effectively, these methods and methodological underpinnings would need to be fully supported by a knowledgeable TQR or qualitative lead (QL).

Methods of integration of qualitative data with other types of data in trials can also be misleading or under-reported. In the phenomenological interviewing example given above, this might demand an inter-textual triangulation approach that recognizes the strengths of the phenomenological paradigm, alongside the strengths of other more quantitatively oriented paradigms (for example, placing ‘lived experience’ narratives alongside statistically defined patient qualitative of life outputs). When considered together, these different methods may provide a deeper understanding and embellishment than any one paradigm or dataset alone. Inter-textual triangulation appreciates the ability of datasets to corroborate rather than simply illustrate one another [8]. Each dataset is, first and foremost, independent and discrete, but seen in relation to one another, they serve to bridge vital gaps in knowledge and understanding, and provide deeper understanding.

Alternatively, researchers can turn for support in integrating data outputs to tools such as the Method for Aggregating the Reporting of Interventions in Complex Studies (MATRICS; Welsh for ‘matrix’) [9] which was presented in detail in published, quasi-experimental gastroenterological studies [10,11].

Results from the integration of methods and study outputs can be jointly presented in journal articles and study reports as a single publication, (see for example Rapport *et al*. [11]).

### Rationale for a qualitative-methods standard operating procedure

Registered clinical trials units (CTUs) use standard operating procedures (SOPs) that offer comprehensive guidance on the conduct of all aspects of the trials. The UK Clinical Research Collaboration (UKCRC) specifies the SOPs expected of all registered CTUs. SOPs have been written for general health [12] and clinical and epidemiological [13] research, and for economic evaluation [14]. However, there is currently no SOP, to our knowledge, to guide qualitative research within trials.

Following discussions with trialists across the UK, we identified the potential for having a qualitative-methods SOP (QM-SOP) for trials. The QM-SOP would act as a steer for future trials work, taking researchers in a similar direction through the advice and guidance on offer, and ensuring that qualitative methods sit firmly within a full SOP portfolio. This would allow qualitative methods to be recognized as both legitimate and necessary elements of trials development within clinical or non-clinical trials. This emphasis on the legitimacy and visibility of qualitative methods within an SOP portfolio can help to reinforce the current understanding that qualitative methods are more than simply add-on or solely developmental elements of trials work, but rather fully integrated elements. By developing this QM-SOP, and presenting it alongside the other 29 SOPs, the West Wales Organisation for Rigorous Trials in Health (WWORTH) team sought to emphasize the value of qualitative methods in trials.

WWORTH is the CTU based in the College of Medicine of Swansea University. In establishing the CTU in 2009, core members of WWORTH worked in groups with colleagues across the college and in the two associated National Health Service (NHS) local health boards to develop a portfolio of 30 SOPs. This work underpinned a successful application by WWORTH to the UKCRC for registration as a CTU (currently provisional status). In particular, group members worked with colleagues skilled in qualitative research methods to develop the QM-SOP for trials. The members had wide experience of qualitative health research, which ran alongside wide experience of economic evaluation and measurement of quality of life (QOL) within pragmatic randomized trials and quasi-experimental studies [15-19]. Qualitative methodologists played an integral role in trial design at Swansea University alongside WWORTH members, initiating and developing trials, triangulating complex datasets, and presenting study outcomes. Hence, they recognized the value of this rare style of team-working, combining methods in a ‘whole-system’ approach to study design.

The next section of the paper presents the methods and results of developing the SOP and describes its application in a case study, the Support and Assessment for Fall Emergency Referrals (SAFER) 2 trial. It then discusses the role of a QM-SOP within a portfolio of SOPs.

## Method

WWORTH initially created three SOP groups: trial administration (TA), trial processes, and trial techniques (TT). The third group drafted the QM-SOP, and designed it to support mainly qualitative researchers in developing, conducting, and reporting qualitative components of trials. Over 6 months, the QM-SOP was developed in accordance with the first WWORTH SOP, namely, the SOP on SOPs, which guided the structure and development of all other SOPs in the SOP portfolio. The process began by reviewing the other SOPs and discussing the content of the QM-SOP, including how this might differ from the other SOPs.

Group members contributed their experience of developing other SOPs and personal views of the questions likely to be asked of a QM-SOP; for example, the specific role of the TQR in relation to the CI.

The QM-SOP describes how to design the qualitative component of a trial with input from team members and service-user representatives, and how to conduct the qualitative component in line with principles of good practice. It includes standard procedures for gaining ethical and research governance approval before contacting any participants; training staff to ensure consistency; and seeking additional informed consent from trial participants, including both staff and users. To promote the sustainability of qualitative research within trials, the SOP advocated recruiting staff with qualitative expertise, and retaining them to enhance the resources and profile of the CTU. The SOP discusses the need for a training plan so that people engaged in trials are conversant with qualitative principles, and have had appropriate supervision and support. It concludes with an appendix forming the training log, which lists trainee, trainer, and the dates and nature of the training.

**Figure 1:**
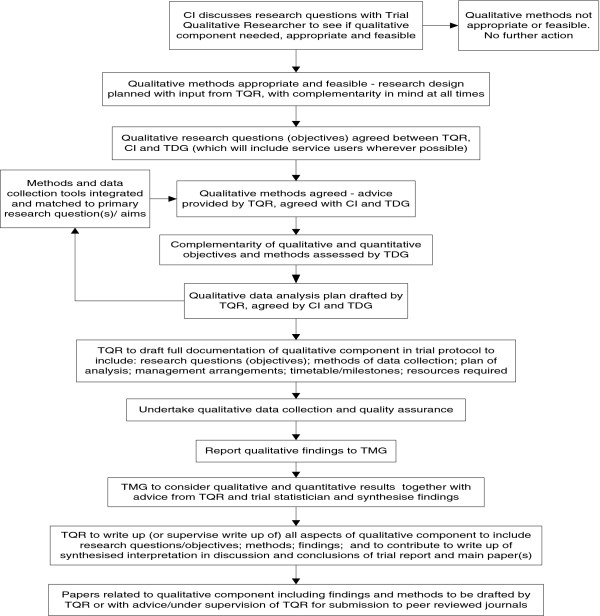
Flow chart of qualitative research within typical trial.

In addition, the QM-SOP is able to support qualitative researchers and methodologists in understanding when and how to clarify the most appropriate methods of data collection and analysis in any one study, and where in the process this should be achieved, along with alignment of methods to research questions. The QM-SOP was designed to be instructive as to the most appropriate time points when these aspects should be fulfilled, with the full agreement of the CI and QL. Discussion points such as these (Figure 1) should fit the overall trial design, but also the manner in which the qualitative research study develops, so that integration of methods is successful. The QM-SOP should direct researchers in hese matters through the TQR and/or the QL, who can advise on the most appropriate methods, in keeping with methodological precepts underpinning qualitative elements (see discussion of this point above).

The SOP group reviewed successive drafts developed initially by two members of the author group, focusing particularly on how well the text reflected past experience and the future needs of those undertaking a range of qualitative techniques in conjunction with a CTU [18]. This process continued until the whole group agreed that the resulting SOP covered the key aspects of qualitative research within trials ,and was consistent with the rest of the WWORTH portfolio of SOPs.

The WWORTH Development Group (WDG) then reviewed the SOP for approval to move from version 0 (‘under development’) to version 1 (‘approved for use’). A designated member of the WDG led the discussion as to whether or not the SOP was ready to move from version 0 to version 1. After the TT-SOP group had addressed any issues identified in this discussion, the WDG formally approved the transition to version 1.

After more than 1 year’s practical experience of using version 1, the WWORTH Joint SOP Group (JSOPG), formed by amalgamating the three original SOP groups, undertook a further review for approval to move from version 1 to version 2 (‘authorized for use’; *see link to approved version*http://www.swan.ac.uk/staff/academic/medicine/rapportf/) of the SOP. Although similar to the first review, the second review sought evidence from three separate trials that had tested version 1 in practice to ensure that it was relevant, accurate, and helpful to new and existing staff, and thus ready for dissemination. The presentation of the SOPs was also streamlined, for example by amalgamating the glossaries of the 30 SOPs in the portfolio into a common glossary.

Within the QM-SOP, an SOP flow chart (Figure 1) was included to enable TQRs to collaborate with other researchers in designing, refining, and timetabling qualitative methods, in keeping with basic trial designs. It was also included to support the development of a qualitative-analysis plan to ensure a smooth transition from data analysis to interpretation, in keeping with other trial methods. The SOP flow chart supported the discussion of qualitative trial aspects between the TQR, the trial CI and the QL.

## Results

In developing the QM-SOP, the authors soon agreed that, although qualitative methods can enhance both conduct and findings of trials, clarity of purpose is essential in the planning stages of the trial. Hence, the QM-SOP stresses the point that mixed methods are most suited to trials whose complexity needs qualitative methods to underpin the quantitative study. If qualitative components have no useful purpose in a specific trial, it is best to focus on the quantitative components alone, or to undertake the qualitative study as a complementary yet separate unit. Consequently, trial protocols must define the qualitative component and its purpose. This should then guide detailed planning, conduct, analysis, and reporting, ideally within the main paper.

The components of the QM-SOP described in this paper comprise the version record, glossary of terms, introduction, purpose, roles and responsibilities of those responsible for implementing the SOP, the procedures themselves, training plan, references, and related WWORTH SOPs. The purpose of the SOP is to describe best practice in designing, conducting, and reporting the qualitative components of trials. The section on roles and responsibilities focuses on people responsible for delivering the qualitative element of trials and putting the SOP into practice, namely the CI, TQR, QL, trial manager/coordinator, and trial data manager. The section on procedures begins with a flow chart of qualitative research (Figure 1) to identify the main steps in developing the qualitative components of a trial. These include assessing whether qualitative methods are appropriate and feasible; definition of questions to be answered using qualitative methods; qualitative methods of data collection; the qualitative-analysis plan; quality assurance; reporting of qualitative findings; and synthesis with other findings.

The original SOP group found it easy to discuss aspects of trial design and conduct relevant to qualitative evaluation without noticeable discord. As they had no previous QM-SOP on which to base development, they had to be innovative in both approach and thinking. As there were thus few restrictions, they worked constructively together to achieve version 1 of the SOP.

Although more constrained by version 1, the later JSOPG also worked smoothly to derive Version 2 of the SOP *[link here to approved version*http://www.swan.ac.uk/staff/academic/medicine/rapportf/*]*. They were keen that the QM-SOP fitted the trials already taking place within WWORTH, including trials in mental health, gastroenterology, and emergency care [17-19]. They also recognized that, although current trials should inform the development and future application of the QM-SOP, they should not limit its scope.

### Case study: the SAFER 2 trial

To show the use of the QM-SOP in practice, this section describes its effective application in a trial in emergency care: SAFER 2, which is currently under way. We describe the trial, and report how it drew on the SOP and benefited from its guidance.

SAFER 2 is a pragmatic randomized trial with a qualitative strand, funded by the National Institute for Health Research, and developed and conducted in line with WWORTH SOPs, including the QM-SOP. It aims to estimate benefits and costs for patients and the NHS of new clinical protocols that enable paramedics to assess older people who have fallen, and refer them to community-based care when needed. SAFER 2 forms part of a program of studies examining the potential, in appropriate circumstances, for paramedics to make safe decisions to use alternative-care pathways rather than transport patients to hospital emergency departments. The first study in the program has already reported, under the name SAFER 1 [19], SAFER 1 was planned before the development of the QM-SOP, but the research team behind SAFER 2 was able to draw on the SOP to shape the study.

The first two objectives of the SAFER 2 study are concerned with the effects of the intervention on patient care, outcome, and costs, and lend themselves to quantitative approaches to data collection and analysis. The second and third objectives are fundamentally qualitative in nature, and seek to understand how patients experience the new health technology, and to identify factors that facilitate or hinder the use of the intervention. The TQR took a lead role in shaping these objectives, and worked with colleagues to ensure that, together with the quantitative objectives, they formed a coherent and integrated whole. The TQR then took on the task of designing and managing the qualitative aspects of the study: planning the data-collection methods and timetable, designing interview schedules and focus-group topic guides, and planning analysis of the qualitative data. After the initial analysis phase, the qualitative and quantitative strands of the study will be re-integrated, so that in the final stages of analysis and writing, two complementary sets of learning will be reported together. The qualitative findings will help to explain why the intervention was or was not successful. They should throw light on the likelihood of the intervention having an effect under ‘real world’ (that is, non-trial) circumstances, through bringing an understanding of how patients and professionals respond to it.

## Discussion

In this paper, we have described the contribution of a QM-SOP to the SOP portfolio of a registered CTU. We believe that using qualitative methods alongside essentially quantitative trials can enhance their design, conduct, and findings. In our experience, combining these distinct paradigms and their associated epistemologies and methodologies benefits each by narrowing the gap between these two approaches, which are often seen as diametrically opposed. This potential for convergence is the main strength of the new SOP. Members of the CTU team learned about the strengths and weaknesses of distinct paradigms and their associated methodologies, by entering into conversation about what each could offer the other. For example, health economists, trialists, and gastroenterologists in the“**E**valuating I**N**novations **I**n **G**astroenterology by the NHS **M**odernisation **A**gency” (ENIGMA) ENIGMA stands for study [10] all took part in elements of the qualitative group analysis, to understand the rich detail of interviews with health professionals and patients. They also took part in several group events that clarified the working practices of the qualitative methodologist and QL, and as a result, understood the qualitative datasets more clearly, which had an effect on both health-economic and statistical analyses. They could describe and define patient QOL issues, and measure them in accordance with pre-defined QOL measures, and they could access knowledge about patient distress at a different level than would otherwise have been the case. This ‘coming together’ of methodological groups, through greater respect of different paradigms, enhanced a mutual understanding between practitioners of different approaches to healthcare research.

Each SOP guides the conduct of a separable element of trials. Our QM-SOP guides the development, management, monitoring, and delivery of qualitative techniques drawn from a rich body of work rarely used in trials. In particular, it defines lines of responsibility and a coherent approach to both planning and reporting. In this way, we seek to consider quantitative alongside qualitative methods and findings; the latter are often perceived as less valuable in reports of trials of complex interventions (for more information on this aspect of integration please see Williams *et al.*[10]; or information on embedding qualitative approaches in quantitative frameworks see Snowdon *et al.*[20]; and for information on integration of qualitative work in systematic reviews see Thomas *et al.*[21]).

### Theory and standard operating procedures: the value of foundational work

The Medical Research Council guidelines for developing and evaluating complex interventions, which underpin many of WWORTH SOPs, give priority to the role of theory [4]. We see theory as the foundation for developing both trials and SOPs. Theoretical ideas inform development of research, researchers’ ability to envisage the creative endeavor, and the relationship between analysis as process and analysis as a means of developing new theory [22]. Coffey and Atkinson describe this as ‘generalizing and theorizing’ ([23], p. 139) and ‘interweaving analysis with the use of ideas’ ([23], p. 140).

Those who analzse trials need to understand theories relevant to both epistemology and the essence of the topic under study. For example, theories of how clinicians interact with patients inform the perceived likelihood of patients complying with advice, and thus influence the design of interventions, the choice of outcome measures, and the analysis methods. If qualitative theory underpins the qualitative element of a trial, it can help to ensure methodological rigor in all aspects of that trial [24]. It can clarify the relationship between the theoretical precept and the qualitative method, and the relationship between the theoretical precept and the study question. For example, continuing with our phenomenological example given earlier, a hermeneutic phenomenological stance can be linked to a research question about lived experience, and a hermeneutic data collection and analysis approach, such as hermeneutic interviewing alongside van Manen’s ‘selective’ or ‘highlighting’ analysis approach [7].

### Research methods and standard operating procedures

Because SOPs give comprehensive guidance on the conduct of trials, our QM-SOP advises on study design and describes the available range of valid methods for collecting and analyzing qualitative data, rather than prescribing methods for each trial. Valid sources of data include documents, focus groups, interviews of all types, and observation [25-27]. Valid methods of analysis include content, conversation, framework, narrative, summative, and thematic [25-27]. The SOP proposes that trial protocols provide rationales for each method chosen. When there is more than one method used, the trial protocol should describe how these together will enhance the study. Although using a mixture of methods may add corroborative insights, it makes ‘mapping’ (considering one dataset alongside another) difficult [25] (p. 121). It is important to plan mapping in advance to avoid one method undercutting another [26], which should therefore yield greater understanding in depth. At its best, mapping should compare datasets by giving each dataset the correct weighting based on its strengths and weaknesses [28].

### Trial monitoring and standard operating procedures

Progressively clarifying mutual understanding also helps in strengthening working relationships, encouraging complementary working practices, and even monitoring trials (the subject of another WWORTH SOP). An effective form of monitoring lies in progress reports to trial-management meetings. These bring all aspects of trial methods to the attention of the management group at regular intervals. Hence, members can better understand the range of methods and the scope for conflict between methods and interpretations, and in triangulating findings. This encourages them to revisit methods and data, identify when and how issues may arise, and even clarify misunderstandings. Thereafter, even differences of interpretation can enhance understanding and reinforce the rigorous process of working in trials.

### Discordant results and standard operating procedures

Qualitative methods in trials can yield understanding in depth of the topic under study by explaining or corroborating outputs from other methods. The sensitive and appropriate use of such triangulation can validate results gained from other aspects of the trial [22,23]. Nevertheless, qualitative results are often inconsistent with quantitative results, or at least need clarification, even when both have followed SOPs. It is therefore important to recognize that qualitative research, with its ability to clarify by adding depth, has a useful role in addressing conflict [24]. Qualitative research can also enable researchers to interpret results through further negotiation [24,25]. These interpretative activities can occur throughout the trial, not only at the start and end, but also during progression from one stage to another. Qualitative data even have a valid role in providing evidence to intervene in the trial if necessary [24].

Thus, it is important for trial teams to work closely as the trial develops. By appreciating the value and nature of this symbiotic relationship, they will be better equipped to understand and deal with divergence when it arises [25]. Moffatt and colleagues [28] stated that, when researchers understand the range of data pathways and links between them, it is easier to scrutinize methodological rigor across the whole study. Indeed, recognizing whether emerging findings are corroborative (without threatening blindness) encourages trialists and methodologists to clarify effects.

## Conclusion

Developing a QM-SOP has enabled this registered CTU to recognize and exploit the potential for qualitative research to underpin, complement and enhance randomized trials. Including qualitative methods in trials that have traditionally been solely reliant on quantitative methods has enriched our understanding of questions addressed by many recent trials.

We recommend that CTUs consider developing a QM-SOP to guide the growing use of qualitative methods alongside trials, and ensure that methods are rigorous, appropriate, and well reported. We conclude that adding qualitative data and analysis improves the design, conduct and reporting of randomized trials, and contributes to the future development of multi-method trials.

## Abbreviations

CI: Chief investigator; CTU: Clinical trials unit; ENIGMA: **E**valuating I**N**novations **I**n **G**astroenterology by the NHS **M**odernisation **A**gency; MATRICS: Method for Aggregating the Reporting of Interventions in Complex Studies; NHS: National Health Service; QL: Qualitative lead; QM: Qualitative methods; QOL: Quality of life; SAFER: Support and Assessment for Fall Emergency Referrals; SOP: Standard operating procedure; TA: Trial administration; TQR: Trials qualitative researcher; TT: Trial techniques; UKCRC: UK Clinical Research Collaboration; WWORTH: West Wales Organisation for Rigorous Trials in Health; WDG: WWORTH Development Group

## Competing interests

All authors declare that they have no competing interests.

## Authors’ contributions

FR, who leads qualitative health research at Swansea University College of Medicine, reviewed the content of the QM-SOP,l and led the writing of this paper. As quality assurance officer of WWORTH, MS managed the review of consecutive versions of the QM-SOP. AP had extensive involvement in writing drafts and reiteration of the paper. HS chaired the WWORTH TT-SOP group and led the development of the QM-SOP. KJ contributed to the WWORTH TT-SOP group and this paper. JP coordinated the TT-SOP group and contributed to this paper. AP and AS coordinated the development of the QM-SOP and contributed to this paper.. SS chaired the WWORTH Joint SOP Group and contributed to the QM-SOP. As acting manager of WWORTH, KT managed the development of its portfolio of SOPs. CC commented on the design and content of the paper as TQR. As director of WWORTH, IR led development of its portfolio of SOPs. All authors commented on successive drafts of this paper, and all authors have read and approved the final manuscript.
